# Examining the validity of the Mini‐Mental State Examination (MMSE) and its domains using network analysis

**DOI:** 10.1111/psyg.13069

**Published:** 2023-12-22

**Authors:** Quoc Cuong Truong, Matti Cervin, Carol C. Choo, Katya Numbers, Adam C. Bentvelzen, Nicole A. Kochan, Henry Brodaty, Perminder S. Sachdev, Oleg N. Medvedev

**Affiliations:** ^1^ University of Waikato School of Psychology Hamilton New Zealand; ^2^ Department of Clinical Sciences Lund, Child and Adolescent Psychiatry, Faculty of Medicine Lund University Lund Sweden; ^3^ College of Healthcare Sciences James Cook University Townsville Queensland Australia; ^4^ Centre for Healthy Brain Ageing (CHeBA), School of Psychiatry University of New South Wales Sydney New South Wales Australia

**Keywords:** cognitive domains, network analysis, neuropsychological assessments, Mini‐Mental State Examination

## Abstract

**Background:**

The Mini‐Mental State Examination (MMSE) is the most widely used standardised screener for impairments across a range of cognitive domains. However, the degree to which its domains (orientation, registration, attention, recall, language, and visuospatial) capture cognitive functioning measured using standardised neuropsychological tests is unclear.

**Method:**

A longitudinal research design with four biannual assessments over a 6‐year period was used with an initial sample of 1037 older adults (aged above 70 years). Participants completed MMSE and neuropsychological tests at each assessment. Network analysis was utilised to investigate unique associations among the MMSE and its domains and neuropsychological test performance at each time point.

**Results:**

The total MMSE and two of its domains, language and recall, were associated with neuropsychological memory performance. The MMSE orientation, registration and visuospatial domains did not have any unique associations with neuropsychological performance. No stable internal interconnections between MMSE domains were found over time. The association of total MMSE as well as its recall domain with neuropsychological memory performance remained very similar over the 6‐year period.

**Conclusions:**

The present study adds evidence to the validity of the MMSE and supports the clinical usage of the MMSE, whereby the total score is used for screening patients with or without cognitive impairments, with repeated administration to monitor cognitive changes over time, to inform intervention. However, the tool is not able to diagnose the cases for changes in specific cognitive domains and as such, should not replace a complete neuropsychological assessment.

## INTRODUCTION

The Mini‐Mental State Examination (MMSE) is the most widely used cognitive screener.^2^ The MMSE is well‐validated^3^, ^4^ and relatively brief, which is suitable for screening cognitive impairments (e.g. dementia). The MMSE consists of 11 items grouped into six domains of orientation, working memory/registration, concentration/attention, recall, language, and visuospatial.^1^, ^5^, ^6^, ^7^ Previous studies have indicated that the MMSE achieved adequate internal consistency (Cronbach alphas of above 0.71), high test–retest coefficients (ranging from 0.80 to 0.89) and good inter‐rater reliability (0.75).^1^, ^8^, ^9^ It was also validated against more comprehensive cognitive assessments such as the Wechsler Adult Intelligence Scale – Revised (WAIS‐R) verbal IQ (*r* = 0.84) and performance IQ (*r* = 0.51).^1^, ^10^, ^11^, ^12^

More recently, a study conducted by Schmitt and colleagues^13^ showed moderate correlations (0.41–0.49) between the MMSE total score and multiple cognitive domains indexed by the Repeatable Battery for the Assessment of Neuropsychological Status (RBANS^14^). Especially, the MMSE showed higher correlations with RBANS factor related to memory ability (*r* = 0.63).^13^ Although the MMSE is a relatively brief assessment tool, it was originally designed to be a measure of global cognition covering a variety of cognitive domains.^1^, ^6^, ^7^, ^15^ However, to the best of our knowledge, validations of the MMSE against neuropsychological tests have only considered the MMSE total score.^16^

Theoretical frameworks such as the Cattell‐Horn‐Carroll (CHC^17^) theory and the Hierarchical Model of Neuropsychological Functioning^18^ suggest that cognitive abilities can be organised into broad domains, with more specific abilities nested within each domain. These domains include attention/processing speed, language, executive function, visuospatial ability, and memory. Research has shown that these domains are not entirely independent and may share common underlying neural networks.^18^ For example, attention/processing speed and executive function have been linked to the prefrontal cortex, while language processing involves both temporal and frontal regions of the brain. Furthermore, deficits in one domain may affect performance in other domains.^17^ For instance, individuals with executive function deficits may also have difficulties with attention and processing speed, while individuals with language deficits may struggle with both verbal memory and visuospatial ability. Thus, theoretical frameworks and empirical research has established organisation and links between neuropsychological cognitive domains. However, the validity of the MMSE domains is unclear and research on associations between its putative domains and domains captured by neuropsychological test performance is needed. A novel and suitable approach to examine unique associations between the MMSE domains and performance‐based measures of cognitive functions is provided by network analysis.^19^

Network analysis is an advanced statistical method that can provide a clearer understanding of unique associations among measured factors (e.g. cognitive domains/aspects).^19^, ^20^, ^21^ In network analysis, all factors are integrated into a single network, which provides a graphical representation of the network and the relations among the included factors.^22^, ^23^ In a network, factors/variables are represented by ‘nodes’ (circles) and relations between variables by ‘edges’ (lines).^24^ The thickness of an edge reflects the strength of each relation. Network analysis has several advantages over more ‘traditional’ methods (e.g. regression and mediation/moderation analyses). That is, it is more robust to outliers and can capture non‐linear relationships that traditional correlation analysis may miss.^25^ Network analysis also provides a graphic network which includes multiple interactions between nodes/variables simultaneously without assigning dependent and independent variables, making it easier to identify important variables and understand the data's structure.^23^, ^26^

The main aim of this study was to explore unique associations between the MMSE domains and comparable domains captured by neuropsychological test performance using a large 6‐year longitudinal sample of older adults. We would also examine unique associations between the total MMSE score and neuropsychological test performance. To our knowledge, there has been no previous studies on the validity of the MMSE domains, thus we would use a combination of exploratory and confirmatory analyses. First, baseline data (i.e. wave 1) would be used to conduct exploratory network analyses, which would be used to form hypotheses. Then, data collected at three follow‐up biannual waves (i.e. waves 2, 3, and 4) would be used to carry out confirmatory network analyses to explore whether associations found in exploratory analyses are replicated across three follow‐up waves.

## METHOD

### Participants

Participants were from the longitudinal Sydney Memory and Ageing Study (MAS) which included a baseline sample of 1037 older adults aged 70 to 90.^27^ MAS participants were from the eastern suburbs of Sydney, Australia, and had appropriate language abilities to complete psychometric assessments.^27^ The major ethnicity of the MAS participants was European (98%); the remaining sample was 1.1% non‐European and 0.8% not revealed. Participants were interviewed every 2 years from wave 1 to wave 4 (6‐year follow‐up). To be included in the MAS at the baseline assessment (wave 1), participants could not have a current or previous diagnosis of dementia, major psychological or neurological disorder, or progressive malignancy. More detailed information about how participants were recruited and their demographics in the MAS can be found in the article by Sachdev and colleagues.^27^ All participants provided written consent to participate in this study, which was approved by the University of New South Wales Human Ethics Review Committee (HC 05037, 09382, 14 327).

**Figure 1:**
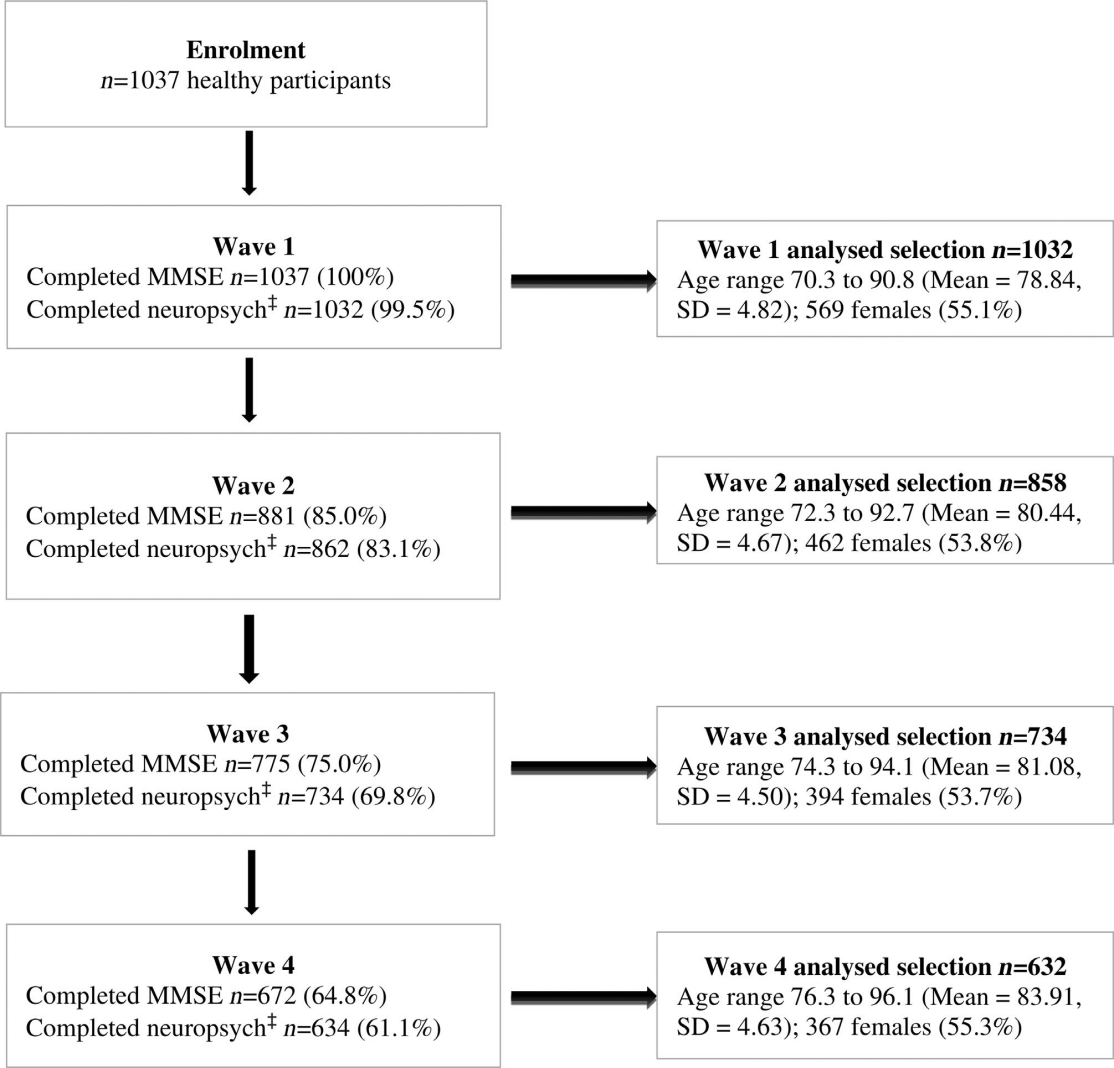
Consolidated Standards of Reporting Trials diagram for participants selected at each wave. MMSE, Mini‐Mental State Examination; SD, standard deviation

Consolidated Standards of Reporting Trials diagram of how participants were selected at each wave for network analyses is presented in Figure 1. Demographic details of participants (i.e. age and sex) at each wave are also displayed in this figure. Independent samples *t*‐tests revealed no significant difference in participant age between females and males across waves (all *P* > 0.05). There were missing data at each wave if participants were not well, had passed away or otherwise unable to complete the assessment at that wave or were not contactable.

### Measures

#### Global cognition assessment

The MMSE^1^ is an 11‐item screening tool designed to capture global cognition and cognitive functions across six domains (i.e. orientation, registration, attention, recall, language, and visuospatial). Individual domain scores are calculated by adding responses of relevant item(s) together.^5^, ^6^, ^7^ The maximum scores for individual domains vary (Supplementary Table S1), for example, the language domain has a maximum score of eight, while the visuospatial domain has a maximum score of one. Total MMSE scores are calculated by summing all MMSE items, with scores ranging from zero to 30.

#### Neuropsychological domains

Performance across five neuropsychological domains (i.e. attention/processing speed, language, executive function, visuospatial ability, and memory) was assessed at wave 1 to wave 4 using a comprehensive neuropsychological battery comprised of 10 tests.^27^, ^28^ The Wechsler Adult Intelligence Scale‐III Digit Symbol‐Coding^29^ and the Trail Making Test A^30^ were used to assess attention/processing speed. The Boston Naming Test^31^ and Animal Fluency^32^ assessed language. The FAS Fluency^33^ and Trail Making Test B^34^ assessed executive function. The Wechsler Adult Intelligence Scale‐Revised (WAIS‐R) Block Design^35^ assessed visuo‐spatial ability. Memory was assessed by the Wechsler Memory Scale‐III (WMS‐III) Logical Memory Story A delayed recall,^36^ Rey Auditory Verbal Learning Test^16^ total learning, short‐term and long‐term recall scores, and the Benton Visual Retention Test.^37^ Composite domain scores were computed for each cognitive domain as follows. Raw test scores were transformed into quasi z‐scores by using baseline means and standard deviations (SDs) from a reference group comprised of 732 MAS participants classified as cognitively healthy at wave 1.^27^ Z‐scores from the neuropsychological tests relevant to each neuropsychological domain were then averaged to compute composite z‐scores for each domain. The exception was the visuospatial domain, which was comprised of a single test. Higher z‐scores indicated better cognitive function.

### Data analyses

Descriptive statistics, including mean, SD, skewness and kurtosis for the domain scores, were computed by IBM SPSS Statistics 28 software. Network analysis was conducted using R software (version 4.0.4; R Core Team, 2021) with the package *BGGM*, short for Bayesian Gaussian Graphical Models.^38^ The Copula Gaussian graphical model estimation was selected to estimate unique associations between nodes because several variables in the study were non‐normally distributed. The networks were illustrated by Gaussian Graphical Models, which graphically present the statistically significant relations between variables in the form of nodes and edges. The nodes represent the variables, and the edges represent unique associations in the form of partial correlations (accounting for all associations among the full set of variables) between nodes and range from −1 (perfect negative association) to +1 (perfect positive association). The strength of each relationship is reflected by the thickness of the edge. Blue lines represent positive relationships and red lines represent negative relationships. For clarity, the MMSE nodes (i.e. total MMSE node and MMSE domain nodes) and the neuropsychological nodes (i.e. attention/processing speed, language, executive function, visuospatial ability, and memory) were assigned different colours. To select which unique relations were statistically significant, we estimated a 95% credible interval (CI) for each association. A CI that excluded zero was considered to indicate a statistically significant edge between nodes. A CI is a range of scores where a specific relation will fall 95% of the time.^39^

The networks were plotted using the R library *qgraph*, which is based on the Fruchterman‐Reingold algorithm.^40^ This algorithm places nodes that are strongly connected with other nodes centrally in the network and nodes with strong connections to each other closely, while avoiding overlap of nodes and edges.^41^ To facilitate comparisons across all four waves in this study, the placement of each node remained the same in all networks by using the averaged network layout, although the inclusion/exclusion (i.e. contingent on whether CIs included zero) and thickness of edges differed.^40^ Strength centrality using the R library *qgraph* was also estimated, which identified how important a node was to each network by counting the number of both negative and positive edges a node had.^26^, ^42^ Strength centrality has been argued to show the most robustness.^23^, ^43^ A node with high centrality has strong links with other nodes in the network and thus acts as a more central node.^42^, ^44^

As introduced, we used a statistical framework that included both exploratory and confirmatory analyses. The exploratory analyses were conducted to explore the network relations between nodes by using the sample of participants at wave 1. These initial analyses allowed us to explore edges between the total MMSE node as well as its domain nodes (i.e. MMSE orientation, registration, attention, recall, language, and visuospatial nodes) and neuropsychological domains/nodes. Post hoc tests were then used (again using wave 1 data) to test whether graphical patterns in the network could be statistically confirmed. True confirmatory analyses were then subsequently conducted using data from the follow‐up waves (i.e. waves 2, 3, and 4) by calculating the posterior probability (PP). A PP indicates the probability of a pre‐specified event occurring, for example, that the edge between nodes A and B is larger than the edge between nodes A and C. PPs above 0.95 (95%) were used as indicators of a confirmed hypothesis in the present study. To calculate PPs, 5000 posterior samples of each edge were estimated and then used for each specific hypotheses (i.e. that the edge between nodes A and B is larger than the edge between nodes A and C).

## RESULTS

### Descriptive statistics

Table 1 presents descriptive statistics, including mean, SD, skewness and kurtosis for the MMSE and neuropsychological nodes across waves. As most MMSE and several other neuropsychological tests had high skewness and/or kurtosis that suggested non‐normal distributions,^45^ Copula Gaussian graphical model estimations were appropriate for estimating associations between nodes.

**Table 1:** Descriptive statistics, including M, SD, skewness, and kurtosis for the domain scores of neuropsychological assessments, and the MMSE on the four waves

	Wave 1				Wave 2				Wave 3				Wave 4			
Scale/domains	M	SD	Skewness	Kurtosis	M	SD	Skewness	Kurtosis	M	SD	Skewness	Kurtosis	M	SD	Skewness	Kurtosis
MMSE																
Orientation	9.50	0.71	−1.36	1.72	9.58	0.77	−2.43	8.36	9.68	0.65	−2.84	8.36	9.63	0.75	−2.93	11.90
Registration	2.97	0.22	−8.31	86.39	2.97	0.18	−6.01	38.25	2.99	0.15	−14.23	242.59	2.97	0.19	−6.03	39.20
Attention	4.85	0.52	−4.30	22.26	4.91	0.37	−5.40	34.83	4.93	0.34	−5.94	39.07	4.89	0.40	−4.19	18.79
Language	7.20	0.72	−0.53	−0.25	7.23	0.81	−0.96	0.79	7.20	0.78	−0.79	0.31	7.09	0.78	−0.70	0.56
Visuospatial ability	0.94	0.24	−3.73	11.94	0.94	0.24	−3.65	11.32	0.95	0.21	−4.27	16.29	0.91	0.28	−2.95	6.74
Recall	2.63	0.61	−1.54	1.90	2.63	0.64	−1.79	2.99	2.62	0.68	−1.91	3.44	2.55	0.74	−1.62	1.89
Total MMSE	28.00	1.51	−0.88	0.99	28.10	1.87	−1.78	5.89	28.04	1.77	−2.12	8.72	27.88	1.92	−1.44	2.39
Neuropsychology																
Attention/processing speed	−0.06	1.04	−0.67	1.71	−0.13	1.21	−1.97	12.94	−0.26	1.21	−1.71	1019	−0.46	1.27	−1.47	6.41
Language	−0.08	1.03	−0.49	0.64	−0.19	1.05	−0.63	0.54	−0.20	1.09	−0.60	0.75	−0.28	1.16	−0.77	0.93
Executive function	−0.06	1.02	−0.57	1.05	−0.18	1.19	−1.32	4.38	−0.19	1.09	−0.69	1.11	−0.41	1.47	−1.54	4.41
Visuospatial ability	−0.04	1.02	0.22	−0.07	−0.01	1.08	0.08	0.01	0.03	1.05	0.11	0.20	−0.18	1.14	−0.01	−0.11
Memory	−0.06	1.01	−0.04	−0.30	−0.11	1.06	−0.01	−0.33	−0.06	1.10	−0.27	−0.20	−0.19	1.17	−0.15	−0.54

Abbreviations: M, mean; SD, standard deviation; MMSE, Mini‐Mental State Examination.

Table 2 displays the means, SDs, and results of statistical comparisons using two‐way repeated analyses of variance (ANOVAs). The independent variables were the scores of MMSE node, its domain nodes, and neuropsychological nodes. The first dependent variable consisted of two subsamples of participants: those diagnosed with dementia after 6 years (*n* = 48) and those who remained healthy/not diagnosed with dementia (*n* = 584). The second dependent variable was the four assessment waves. The results indicate significant interaction effects between subsamples and waves on all neuropsychological nodes (all *P* < 0.001) and the majority of the MMSE nodes (*P* ≤ 0.04), except for the scores of MMSE registration (*P* = 0.37) and language (*P* = 0.84). Post hoc tests revealed that the dementia subsample scored significantly lower compared to the healthy subsample across all waves on all neuropsychological nodes (*P* < 0.001), as well as the scores of MMSE and its recall nodes (*P* < 0.001). However, the MMSE orientation, attention, and visuospatial nodes were only significantly lower in the dementia subsample compared to the healthy subsample at waves 2, 3, and 4. Additionally, all nodes (MMSE node, its domain nodes, and neuropsychological nodes) generally showed a decrease over the 6‐year period within both subsamples. Notably, the decrease was more pronounced in the dementia subsample compared to the healthy subsample. These findings indicate significant differences between changes of cognitive scores over the 6‐year period of those diagnosed with dementia at wave 4 compared to healthy participants.

**Table 2:** Descriptive statistics, including M and SD, and statistical comparisons using repeated ANOVAs (interactional effect *P*‐values) for the domain scores of neuropsychological assessments, and the MMSE on the four waves in the subsample of participants who were diagnosed with dementia after the 6‐year period (*n* = 48) and that of those who were healthy/not dementia (*n* = 584)

Scale/domains	Subsample	Wave 1	Wave 2	Wave 3	Wave 4	*P*
		M (SD)	M (SD)	M (SD)	M (SD)	
MMSE						
Orientation	Dementia	9.35 (0.73)	9.29 (1.01)	9.16 (1.18)	8.25 (1.56)	<0.001
	Healthy	9.54 (0.69)	9.66 (0.66)	9.74 (0.49)	9.72 (0.55)	
Registration	Dementia	2.96 (0.20)	2.96 (0.20)	2.98 (0.15)	2.92 (0.28)	0.37
	Healthy	2.97 (0.21)	2.97 (0.18)	2.99 (0.09)	2.96 (0.21)	
Attention	Dementia	4.73 (0.61)	4.83 (0.63)	4.84 (0.43)	4.45 (0.9)	<0.001
	Healthy	4.85 (0.51)	4.93 (0.30)	4.94 (0.28)	4.90 (0.40)	
Language	Dementia	6.94 (0.81)	6.90 (0.78)	6.84 (0.91)	6.58 (1.09)	0.84
	Healthy	7.27 (0.72)	7.24 (0.82)	7.20 (0.77)	7.07 (0.79)	
Visuospatial ability	Dementia	0.92 (0.28)	0.90 (0.31)	0.91 (0.29)	0.77 (0.42)	0.04
	Healthy	0.93 (0.25)	0.95 (0.22)	0.95 (0.21)	0.91 (0.29)	
Recall	Dementia	2.42 (0.71)	2.23 (0.88)	1.86 (0.98)	1.40 (1.09)	<0.001
	Healthy	2.72 (0.53)	2.71 (0.54)	2.67 (0.58)	2.60 (0.65)	
Total MMSE	Dementia	27.31 (1.70)	27.10 (1.57)	26.59 (2.18)	24.27 (2.70)	<0.001
	Healthy	28.27 (1.40)	28.45 (1.47)	28.50 (1.33)	28.15 (1.56)	
Neuropsychology						
Attention/processing speed	Dementia	−0.54 (0.91)	−0.81 (1.08)	−1.58 (1.79)	−2.47 (1.99)	<0.001
	Healthy	0.10 (0.91)	0.01 (1.03)	−0.15 (1.04)	−0.40 (1.12)	
Language	Dementia	−0.73 (1.06)	−0.99 (1.17)	−1.54 (0.99)	−2.04 (1.18)	<0.001
	Healthy	0.06 (1.04)	−0.08 (1.00)	−0.12 (1.03)	−0.26 (1.09)	
Executive function	Dementia	−0.44 (1.08)	−1.02 (1.55)	−1.29 (1.41)	−2.82 (2.25)	<0.001
	Healthy	0.11 (0.95)	0.01 (0.98)	−0.08 (0.99)	−0.29 (1.27)	
Visuospatial ability	Dementia	−0.42 (0.88)	−0.45 (0.98)	−0.73 (1.03)	−1.39 (1.25)	<0.001
	Healthy	0.12 (1.00)	0.07 (1.05)	0.08 (1.02)	−0.16 (1.07)	
Memory	Dementia	−0.86 (1.02)	−1.24 (0.91)	−1.75 (0.86)	−2.18 (0.56)	<0.001
	Healthy	0.14 (0.96)	0.06 (0.97)	0.06 (1.01)	−0.10 (1.10)	

Abbreviations: ANOVA, analysis of variance; M, mean; MMSE, Mini‐Mental State Examination; SD, standard deviation.

### Exploratory analyses

**Figure 2:**
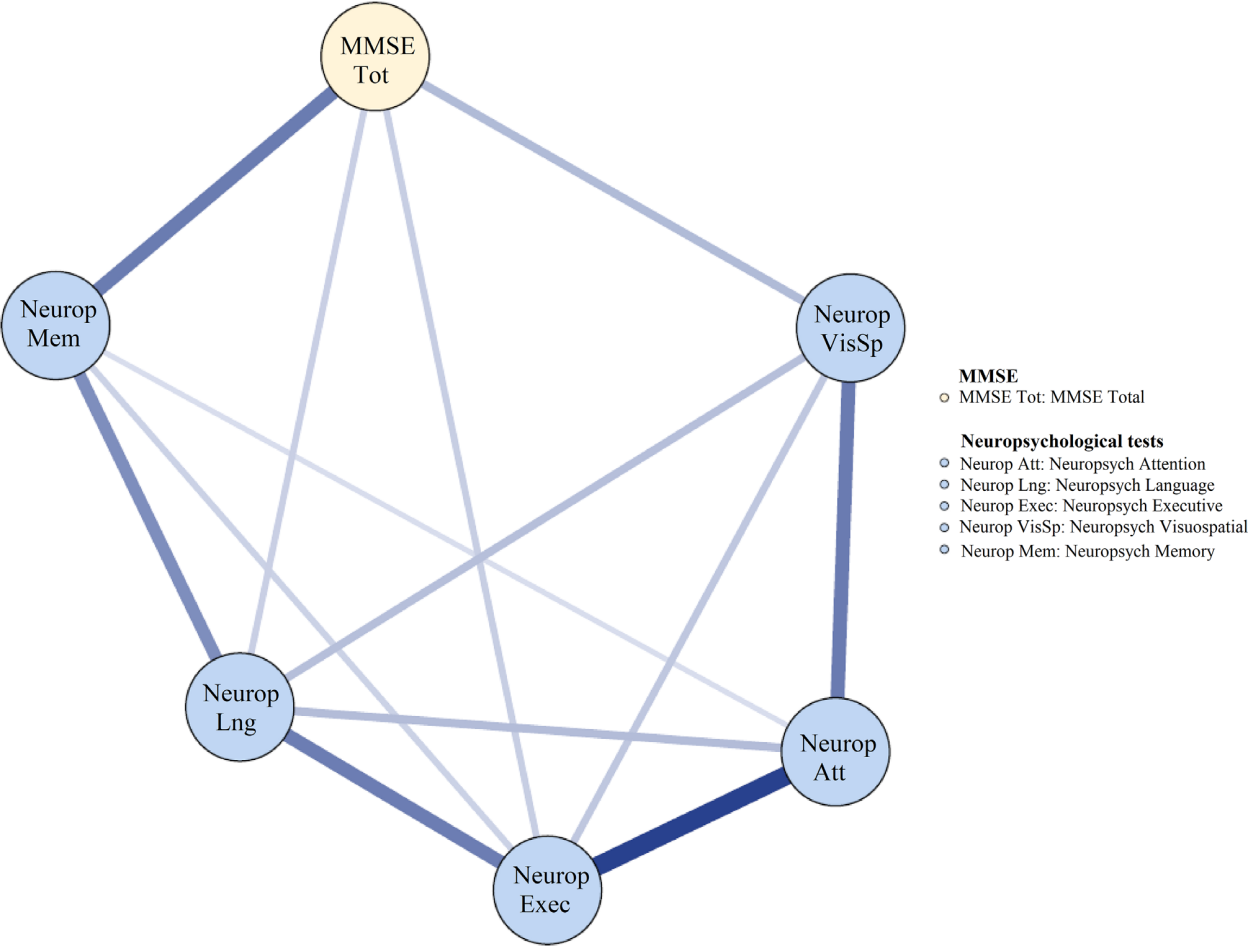
Exploratory network of neuropsychological domains and the total MMSE at wave 1. MMSE, Mini‐Mental State Examination

The exploratory network for the total MMSE node and neuropsychological assessment nodes at wave 1 are presented in Figure 2. The total MMSE node had statistically significant edges to most neuropsychological nodes (i.e. edge weights ranging from 0.34 to 0.42), except for the neuropsychological attention node. However, results from post hoc tests indicated that the edge between the total MMSE node and the neuropsychological memory node was stronger than between the total MMSE and all other neuropsychological nodes (all PPs =100%). It means that only the significant edge between the total MMSE node and the neuropsychological memory node was confirmed.

**Figure 3:**
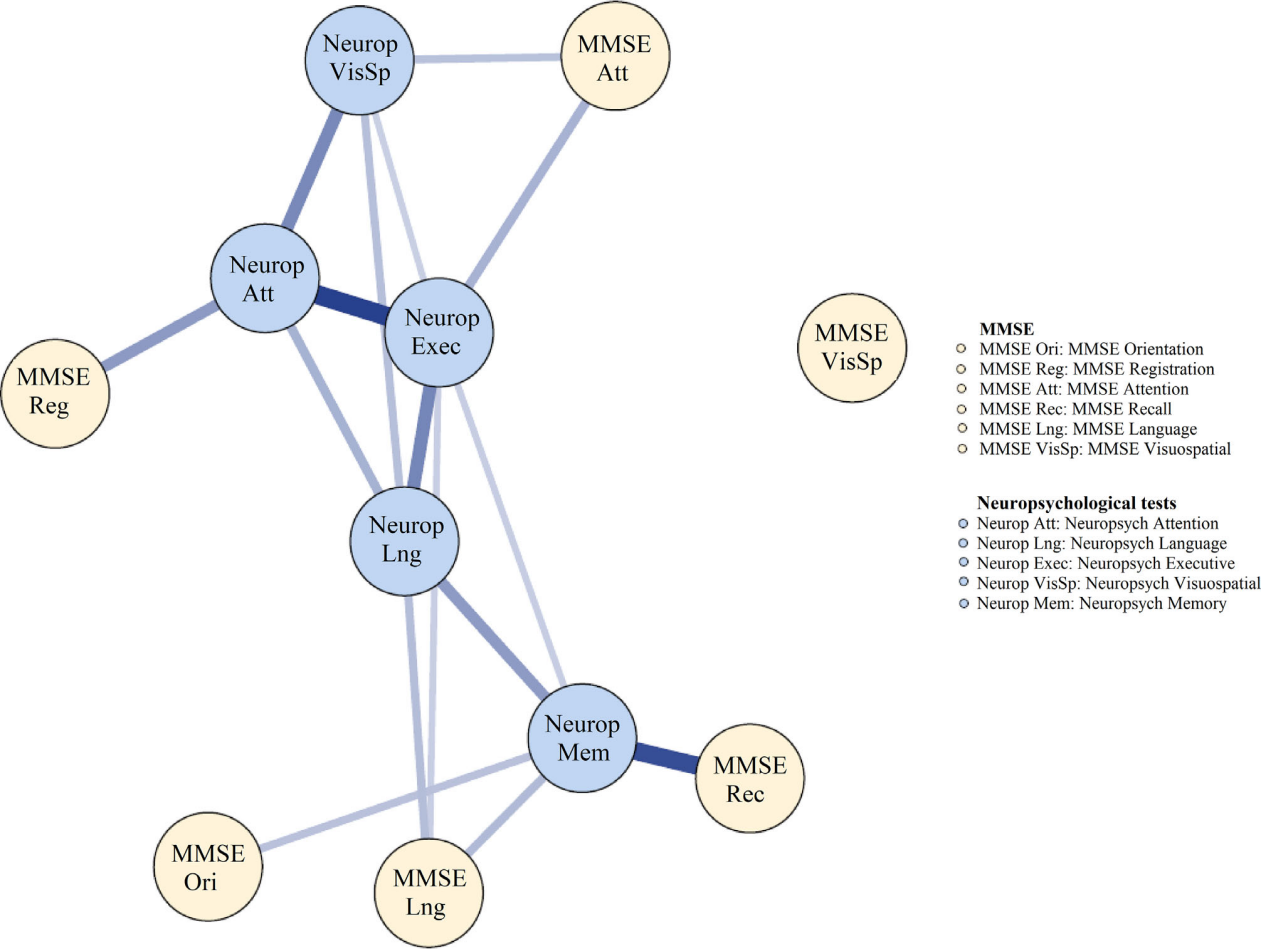
Exploratory network of neuropsychological domains and MMSE domains at wave 1. MMSE, Mini‐Mental State Examination

Figure 3 displays the exploratory network for MMSE domain nodes and neuropsychological domains/nodes at wave 1. The neuropsychological nodes showed many edges between nodes internally were statistically significant (i.e. edge weights ranging from 0.27 to 0.59) meaning that they were highly interconnected. Conversely, the MMSE domain nodes were not interconnected to each other with most internal edge weights being close to zero, which means that domains are not sharing common variance as would be expected for cognitive domains based on theoretical and empirical evidence.

The MMSE language node had significant edges with neuropsychological language node (edge weight of 0.29, 95% CI (0.057, 0.213)) and neuropsychological memory node (edge weight of 0.27, 95% CI (0.049, 0.220)). However, post hoc tests based on the posterior probability estimates only statistically confirmed the edge between the MMSE language node and the neuropsychological memory node (all PPs > 96.2%). In addition, the MMSE recall node had a significant edge with the neuropsychological memory node (edge weight = 0.37, 95% CI (0.253, 0.420)) and this edge was confirmed with all PPs >99.9%. Furthermore, the MMSE orientation node had a significant edge with neuropsychological memory node (edge weight = 0.37, 95% CI (0.253, 0.420)) and the MMSE registration node had a significant edge with neuropsychological attention node (edge weight = 0.37, 95% CI (0.253, 0.420)). However, post hoc tests did not confirm these two edges as most PPs were below 95.0%. Moreover, Figure 3 also shows no associations between the MMSE visuospatial node and other neuropsychological nodes. Furthermore, centrality of nodes was also estimated at this wave, which is presented in Supplementary Figure S1. As can be seen, all neuropsychological nodes had extensively higher centrality compared to all MMSE nodes. This suggests that all neuropsychological nodes reflected cognitive abilities that were more influential at wave 1.

In summary, our exploratory network results allowed us to form hypotheses that: (i) there would be a link between the total MMSE node and the neuropsychological memory node; (ii) there would be no links between the MMSE nodes internally; (iii) the MMSE language node would be more strongly related to neuropsychological memory node than to the other neuropsychological nodes; (iv) the MMSE recall node would be more strongly related to neuropsychological memory node than to the other neuropsychological nodes; (v) the MMSE orientation, registration and visuospatial nodes would not have any associations with any of the neuropsychological nodes; and (vi) the neuropsychological nodes would be more central or influential in comparison to the MMSE nodes on the network. Next, confirmatory analyses using data collected at waves 2–4 were conducted to test these hypotheses.

### Confirmatory analyses

**Figure 4:**
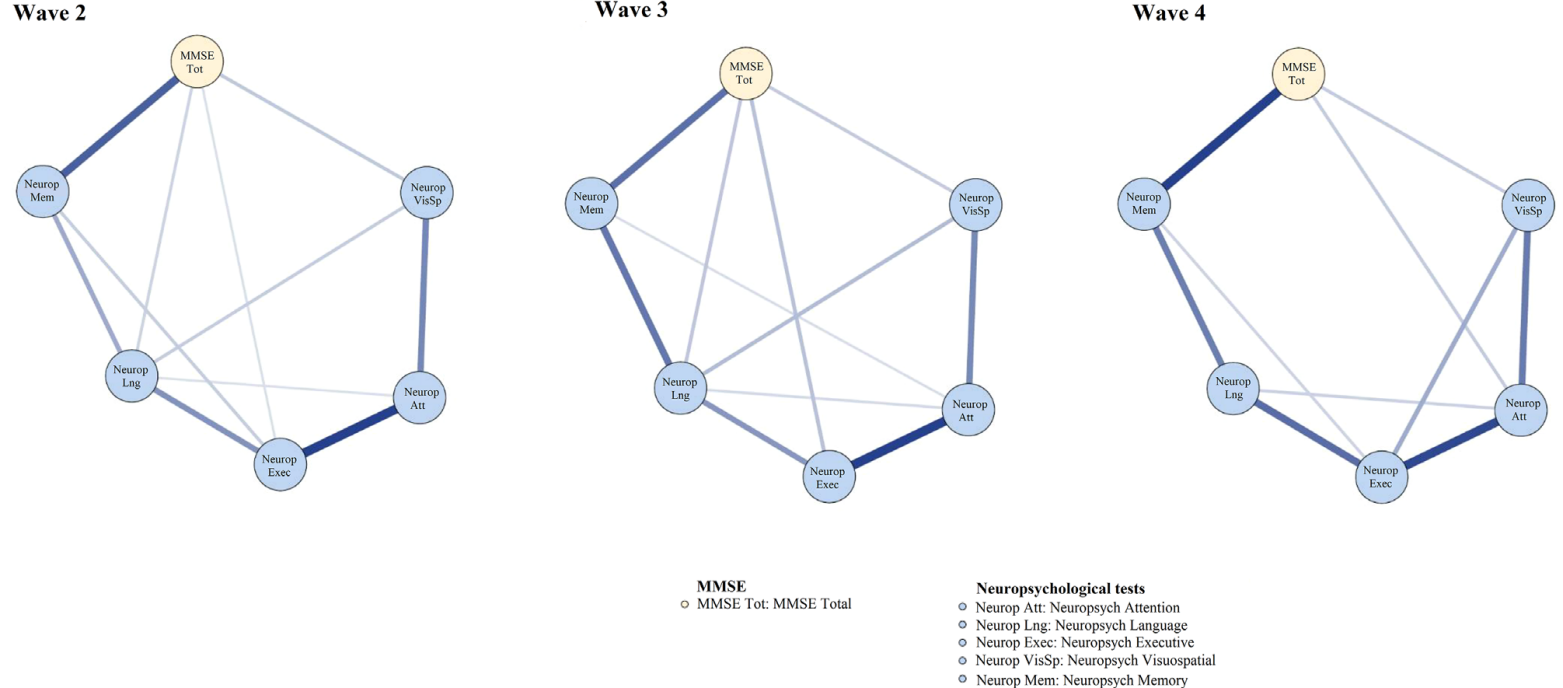
Confirmatory networks of neuropsychological domains and the total MMSE at follow‐up waves. MMSE, Mini‐Mental State Examination

**Figure 5:**
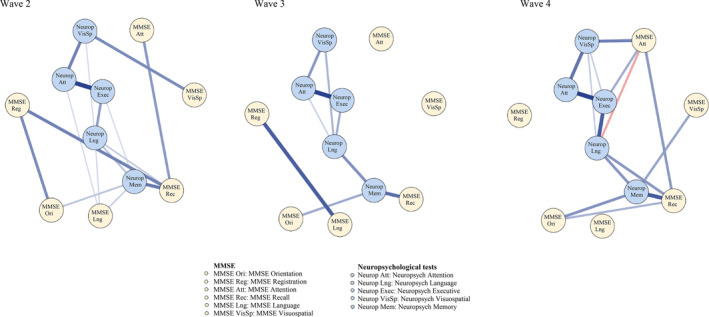
Confirmatory networks of neuropsychological domains and MMSE domains at follow‐up waves. MMSE, Mini‐Mental State Examination

Figures 4 and 5 present the estimated networks with averaged network layouts for the total MMSE node, as well as its domain nodes, and the neuropsychological nodes across the three follow‐up waves. As shown, the networks for follow‐up waves only partly confirmed our hypotheses formed at wave 1. More specifically, hypothesis 1 that the links between the total MMSE node and the neuropsychological memory node was confirmed in waves 2–4. Second, hypothesis 2 that there would be no links between the MMSE nodes internally was not confirmed in confirmatory analyses; rather, while MMSE nodes were internally associated across waves 2–4, these MMSE internal associations were unstable over time. Third, hypothesis 3 that the MMSE language node would be more strongly related to neuropsychological memory node as compared to the other neuropsychological nodes was not confirmed across waves as there were no associations between the MMSE language nodes and the neuropsychological memory nodes at waves 3 and 4. However, hypothesis 4 was confirmed across waves as the association between the MMSE recall node and the neuropsychological memory nodes remained unchanged over the 6‐year period.

Furthermore, hypothesis 5 was not confirmed across follow‐up waves as significant associations between the MMSE orientation, registration and visuospatial nodes emerged in regard to other neuropsychological nodes across waves. For example, there were significant associations between the MMSE visuospatial node and the neuropsychological visuospatial node at wave 2, and between the MMSE orientation node and the neuropsychological memory node at wave 3. Lastly, hypothesis 6, that the neuropsychological nodes would be more influential in comparison to the MMSE nodes on the networks, was confirmed. In fact, most links between the neuropsychological nodes internally remained stable and most neuropsychological nodes had extensively higher centrality compared to all MMSE nodes (see Supplementary Fig. S1) across waves 2–4.

In summary, the confirmatory results indicated that three out of six hypotheses, including hypothesis 1, 4 and 6, were fully confirmed across follow‐up waves.

## DISCUSSION

The present study applied network analysis in a novel way to investigate the validity of a standardised cognitive screener, the MMSE, in a large ageing sample using longitudinal data from four assessments collected over a 6‐year period. Overall, the total MMSE score was consistently associated with the neuropsychological memory node over time. In other words, our finding revealed that the total score of the MMSE appears to capture mostly the memory aspect of neuropsychological test performance. This is in line with previous research that the correlation between the MMSE and memory function captured by comprehensive neuropsychological tests was higher compared to that between the MMSE and other cognitive abilities.^13^ This indicates that the MMSE will continue to be the common and first‐line screening tool for cognitive impairments in older adults because memory loss is commonly used as a criterion to detect abnormal cognitive ageing conditions such as dementia.^46^

However, no robust relations between the individual MMSE domains and the neuropsychological domains appeared over the same period (6 years). Moreover, the individual neuropsychological domains showed consistent internal edges (i.e. significant associations) across waves, but the six domains of the MMSE were not consistently linked to each other internally across the four waves. The links found between the MMSE domains lacked stability over time and most of them (except the MMSE recall domain at waves 2 and 4) had considerably lower centrality within each wave as compared to the coherent and stable internal relations of the neuropsychological nodes. Since the MMSE is a brief cognitive screener, its individual domains might not represent cognitively comprehensive constructs, especially given each domain is comprised of very few items, and hence its domains cannot share common variance as would be expected for cognitive domains based on theoretical and empirical evidence.^17^, ^18^ For example, the MMSE visuospatial domain had only one item requiring participants to copy a pentagon, which was only a proxy measure of visuospatial function rather than adequately representing the actual skill. Therefore, the MMSE domains should be interpreted cautiously, which is in line with previous recommendation that the domains of the MMSE are not empirically derived and may not be valid indicators of specific cognitive domains.^16^

It is noteworthy that the association between the MMSE recall domain and neuropsychological memory domain remained consistent over the 6‐year period. The other associations between the MMSE and neuropsychological domains found in wave 1 tended to change over time. In the same way, links between the MMSE domains changed across waves. For example, there were significant internal edges between MMSE registration, orientation, and recall domains at wave 2. However, these edges were changed at the followed‐up waves (i.e. wave 3 and 4). At wave 3, the only internal association between MMSE domains that reached significance was between registration and language. At wave 4, two internal MMSE associations emerged between orientation and recall, and between attention and recall. These appear to confirm that the MMSE is capturing cognitive function that is sensitive to change over time, which is consistent with previous findings.^47^

It should also be noted that the interconnections between neuropsychological domains remained stable over most waves. This result is consistent with theoretical frameworks^17^, ^18^ and in line with a recent literature reviews.^48^ These data add more empirical evidence that neuropsychological domains, including attention/processing speed, language, executive function, visuospatial ability, and memory, may share features.^18^ This finding also contributes to the body of knowledge supporting Spearman's two‐factor theory of intelligence that suggests overarching general intelligence (‘g’) captures specific abilities (‘s’) in older individuals.^49^ Specifically, high centrality of all neuropsychological domains and stable links between the domains support the theory that older people with higher overall ‘g’ – or high general intelligence – are more capable of other cognitive abilities. At the same time, different associations found between distinct domains support the presence of ‘s’ abilities in older populations as well.

Moreover, our results indicate differences in score shift patterns between the healthy and dementia subsamples. This shows that the progression to dementia adversely influenced the cognitive performance of participants, as captured by the MMSE and neuropsychological tests. Therefore, this finding enhances our understanding of the significance of identifying potential differences that could highlight early signs of cognitive decline and, ultimately, dementia. However, these results should be interpreted cautiously as we experienced a high attrition rate in our sample size, with only 60% of participants having data for all four waves. This attrition may have impacted the statistical power of the results.

Our study had some limitations, which should be acknowledged. Participants were predominately White, European, and well‐educated and recruited from an affluent area of Sydney, Australia, and hence our sample is not representative of the general population of older adults or specifically older Australian adults. This may bias the results of neuropsychological tests. Therefore, future studies should replicate this study with more diverse and representative samples of older adults. In addition, the confirmatory analyses were conducted entirely within the same sample that was used to derive our initial hypotheses (although at different time points). Confirmatory tests in true independent samples would have been a stronger source of evidence and should be explored by future studies.

In conclusion, the MMSE is a commonly used standardised cognitive screener that systematically assesses cognitive function, including: orientation, registration, attention and calculation, recall, and language. The results from the present study add evidence to the validity of the MMSE and support the clinical usage of the MMSE whereby the total score is used for screening patients with or without cognitive impairments, with repeated administration to monitor cognitive changes over time, to inform intervention. However, the tool is not able to diagnose the cases for changes in specific cognitive domains and as such, should not replace a complete neuropsychological assessment.

## DISCLOSURE

The authors have no potential conflicts of interest to disclose.

## COMPLIANCE WITH ETHICAL STANDARDS

The study complied with the guidelines of the university ethics committee, which are internationally accepted ethical standards.

## Supporting information


**Figure S1.** Centrality plots from exploratory and confirmatory networks of neuropsychological domains and Mini‐Mental State Examination (MMSE) domains across four waves.

**Table S1.** Domains, individual items and maximum score of items of the Mini‐Mental State Examination (MMSE).

## Data Availability

The data that support the findings of this study are available on request from the corresponding author. The data are not publicly available due to privacy or ethical restrictions.
