# Supplementary figures and images for: Examining the validity of the Mini‐Mental State Examination (MMSE) and its domains using network analysis
Source: Psychogeriatrics. 2023 Dec 22;24(2):259–71. doi: 10.1111/psyg.13069 (PMC11577997; doi:10.1111/psyg.13069)

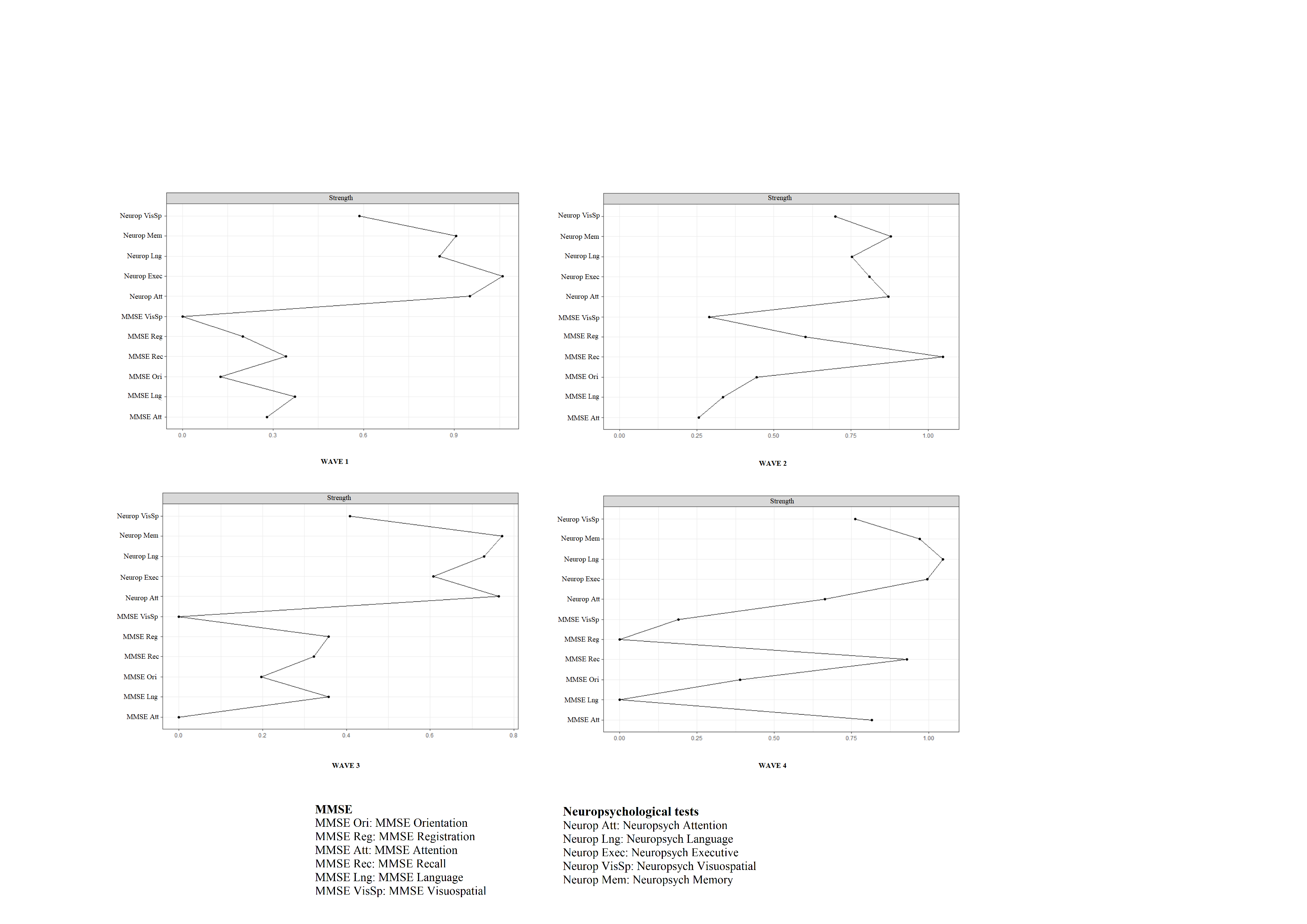

Supplement: Supplementary file 1 — Figure S1. Centrality plots from exploratory and confirmatory networks of neuropsychological domains and Mini‐Mental State Examination (MMSE) domains across four waves. [file PSYG-24-259-s001.png]
