# Supplementary material for: Examining the validity of the Mini‐Mental State Examination (MMSE) and its domains using network analysis
Source: Psychogeriatrics. 2023 Dec 22;24(2):259–71. doi: 10.1111/psyg.13069 (PMC11577997; doi:10.1111/psyg.13069)
Supplement: Supplementary file 2 — Table S1. Domains, individual items and maximum score of items of the Mini‐Mental State Examination (MMSE). [file PSYG-24-259-s002.docx]

| **Supplementary** **Table S1**  *Domains, individual items and maximum score of items of the MMSE* | | |
| --- | --- | --- |
| **Domains** | **Individual items** | **Max item score** |
| Orientation | 1. What is the year? Season? Date? Day? Month? | 5 |
|  | 2. Where are we now? State? County? Town/city? Hospital? Floor? | 5 |
| Registration | 3. Listen to the names of three objects and then recall them | 3 |
| Attention | 4. Subtract 7 from 90 and keep subtracting 7 from each new number (If there are one or more errors on the serial 7s then ask participants to spell world backwards) | 5 |
| Recall | 5. Recall three objects read out earlier | 3 |
| Language | 6. What is this called? | 2 |
|  | 7. Repeat a phrase: “No ifs, ands, or buts” | 1 |
|  | 8. Take this piece of paper in your (non-dominant) hand, fold it in half using both hands, and put the piece of paper on the floor | 3 |
|  | 9. Read the words on this page and then do what it says | 1 |
|  | 10. Write any complete sentence | 1 |
| Visuospatial | 11. Copy this design (pentagons) | 1 |
